# A Key to New World *Distatrix* Mason (Hymenoptera: Braconidae), with Descriptions of Six New Reared Neotropical Species

**DOI:** 10.1673/031.009.2901

**Published:** 2009-06-02

**Authors:** Christopher C. Grinter, James B. Whitfield, Heidi Connahs, Lee A. Dyer, Winifred Hallwachs, Daniel H. Janzen

**Affiliations:** ^1^ Department of Entomology, University of Illinois, Urbana, Illinois, 61801 USA; ^2^ Department of Ecology and Evolutionary Biology, Tulane University, New Orleans, LA 70118 USA; ^3^ Department of Biology, University of Pennsylvania, Philadelphia, PA 19104, USA

**Keywords:** parasitoid, Microgastrinae, Lepidoptera, inventory, *Distatrix loretta*, *Distatrix xanadon*, *Distatrix vigilis*, *Distatrix pitillaensis*, *Distatrix pandora*, *Distatrix antirrheae*

## Abstract

Six new species of the genus *Distatrix* Mason from Central and South America, *D. loretta, D. xanadon, D. vigilis, D. pitillaensis, D. pandora* Grinter, n. sp., and *D. antirrheae* Whitfield & Grinter, n. sp., are described from large-scale caterpillar inventory endeavors, mostly from the larvae of geometrid moths. Biological information and diagnostic features that distinguish these species from other previously described *Distatrix,* especially those from the Neotropical region, are provided; and the first key to New World species is presented. The new discoveries expand our knowledge of the World's widespread *Distatrix* fauna by about a third, suggesting that similar survey efforts in other poorly sampled regions will reveal numerous additional undescribed species.

## Introduction

Six new species of *Distatrix* Mason have recently been reared from biological inventories in the Neotropics. This unusual genus of braconid wasps (Microgastrinae) has often been confused with the superficially similar but much more common and diverse genus *Glyptapanteles* Ashmead, resulting in few reliable records of its distribution and biology. Mason (1981) erected this genus to include members of Nixon's (1965) primarily tropical *formosus* —group of *Apanteles* Förster, and suggested (based on the shared miniaturization of the ovipositor sheath setae) that the genus might be related to another of his newly erected genera, *Rasivalva* Mason. More recent combined analyses of molecular and morphological data (Whitfield et al. 2002) suggest that its closest relative is more likely to be *Venanides* Mason, which it more closely resembles in other body features. Unfortunately all three genera are still relatively rare in collections and have been little studied biologically until recently.

*Distatrix* has been known in the New World only from southern Mexico (Nixon 1965) and California (Whitfield & Scaccia 1996), although Whitfield et al. (1999) reported an additional, probably undescribed but unfortunately poorly preserved, species reared from *Hyperstrotia secta* (Grt.) (Noctuidae) on Missouri Ozark oaks. Thirteen other species have been described worldwide (keyed in Nixon 1965); these are distributed throughout Africa and Eurasia but nowhere appear to be very diverse. The species have been sporadically reported from a wide spectrum of macrolepidopteran hosts (although most often Geometridae and Noctuidae), and exhibit a broad range of both solitary and gregarious cocoon types, including the pedunculate type exhibited by most of the new species described below. The known members of the genus will usually key successfully in Wharton et al. (1997) to *Distatrix.*

Four of the six new species were reared from specimens collected for the caterpillar and parasitoid inventory of the Area de Conservación Guanacaste (ACG), north-western Costa Rica (http://janzen.sas.upenn.edu) (*D. loretta, xanadon, vigilis, pitillaensis*). Each of these species comes from a distinct ecosystem, ranging from 285 meters (*D. vigilis*) to 1220 meters (*D. xanadon*), and has a specific and different caterpillar host species. Since the establishment of the ACG inventory there have only been seven specimens of *Distatrix* reared from approximately 390,000 wild-caught caterpillars (Janzen pers. comm.). A leg from each of these specimens has been DNA barcoded by the BOLD initiative (http://www.barcodinglife.org) (labeled with DHJPAR#). Of the six specimens sampled only four have produced viable DNA (one remaining in queue in Canada awaiting COI sequence, the final species produced poor sequence quality). Distance based analysis of the sequences show substantial nucleotide differences (6–18%, M.A. Smith, pers. comm.), that in conjunction with diagnostic morphological and biological characters, establishes new species status (Hajibabaei et al. 2007).

The remaining two new species were reared from the Caterpillars and Parasitoids of the Ecuadorian Andes project (www.caterpillars.org) and by related rearing projects in Panama (Barro Colorado Island) and Costa Rica (La Selva Biological Station (Dyer & Gentry 2002)). While *Distatrix pandora* is known from all three locations in relative abundance (especially in Central America), *Distatrix antirrheae* has only once been reared from eastern Ecuador. This second species of *Distatrix* is known from the rarely collected Morphinae butterfly, *Antirrhea adoptiva porphyrosticta* (Nymphalidae). This host preference, along with several morphological characters, strongly corresponds to many of the eleven Old World *Distatrix* and is unique amongst all known New World taxa.

Specimens of *Distatrix pandora* were collected in larger numbers due in part to a concentrated search for the host caterpillar *Eois* (Geometridae). Because of the small and cryptic nature of the *Eois* caterpillar (Figure 32) special search routines would be required by other rearing endeavors to produce comparable numbers of parasitoids. In Ecuador, from 2000–2008, we collected over 9,000 individual *Eois* caterpillars (72 species), and reared out over 400 parasitic Hymenoptera and Diptera. This focused collection of *Eois* yielded higher diversity of parasitoids than the assemblage reared from *Eois* in Panama and Costa Rica (L. Dyer, unpublished data) and included multiple species of Braconidae (46% of all parasitoids), Ichneumonidae (27%), Tachinidae (22%), Pteromalidae (2%), and Eulophidae (1%). If *D*. *pandora* is specialized on *Eois* then it is surprising that this rich yield of parasitoids did not include more specimens of *Distatrix.* Nevertheless, based on the high levels of *Eois* parasitism and the high densities of *Eois* (L. Dyer, unpublished data), the Ecuador site will certainly prove to be an important location for future work on *Distatrix-Eois* relationships and the importance of *Distatrix pandora* in geometrid parasitoid communities.

Throughout the world, different species of *Distatrix* parasitize a wide range of hosts: Papilionidae, Nymphalidae, Noctuidae and Arctiidae (Nixon 1965); with a preference for the Geometridae in the New World. Individual species, however, appear to specialize upon closely related genera of lepidopteran larvae. Work emerging from the Ecuadorian and Costa Rican forests is beginning to show unprecedented levels of host-parasitoid specialization (Dyer et al. 2007; Janzen, pers. comm.; Smith et al. in review), that may be evident among the Neotropical *Distatrix.* Further work is necessary to determine the phylogenetic relationships of *Distatrix,* not only amongst the Microgastrinae, but in the context of biogeography and associated lepidopteran hosts.

Below we provide several biological details and morphological notes that will help distinguish all known Neotropical species of *Distatrix.* Terminology follows Mason (1981) for body structures, and Sharkey (1994) for wing venation. *Distatrix* as erected by Mason (1981) possesses, but is not completely limited by, the following characters: absence of the upper pronotal groove; vannal lobe of the hindwing subapically straight to concave, with reduced to nearly absent fringe of hairs; ovipositor sheaths reduced, with minute apical hairs, and the more or less triangular form of the second metasomal tergum. In some species the compound eyes of the female are conspicuously enlarged, so that most of the head is covered by eye. This feature, along with the largely xanthic coloration, suggests that these species are nocturnally active (Whitfield & Saccia 1996). Five of the six known cocoons of the new species also share with *D. formosus* (Wesmael) and *D. solanae* Whitfield the behavior of producing stalked cocoons on nearby substrate of the host.

## Key to the Neotropical species of *Distatrix* Females

### *Distatrix loretta* Grinter, New Species Figures 1–2, 30, 36)

Holotype female. — Body length 2.65mm; forewing length 2.96mm.

***Head*:** black except yellow-orange palpi, honey-orange labrum, mandibles, clypeus and frontoclypeal suture. Scape mottled black and brown, flagellomeres dark brown. Frons barrel-shaped and medially taller than broad due to size of eyes, weakly punctate. Eyes extremely large, covering most of lateral view of head. Interior margin of eyes narrowing slightly towards clypeus then tapering off ventrally. Antennae longer than forewing length and tapering gradually towards apex. Ocelli in an equilateral triangle, distance to eye-margin from lateral ocellus equal to one of its longer diameters. All but distal flagellomeres with two ranks of placodes.

**Table:**
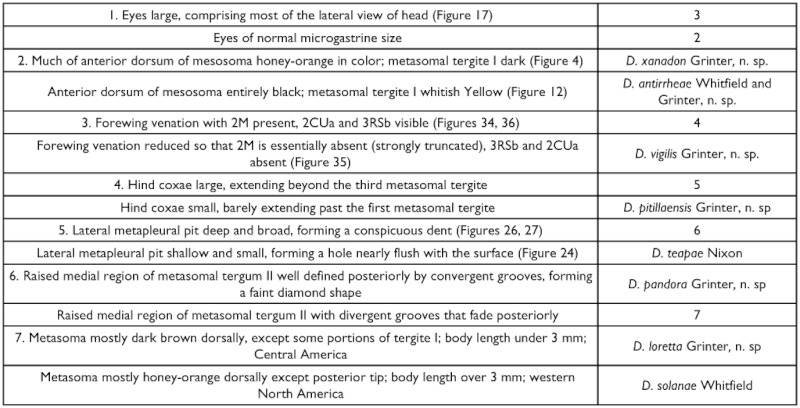
Key to the Neotropical species of *Distatrix* Females

**Figures 1–6.**
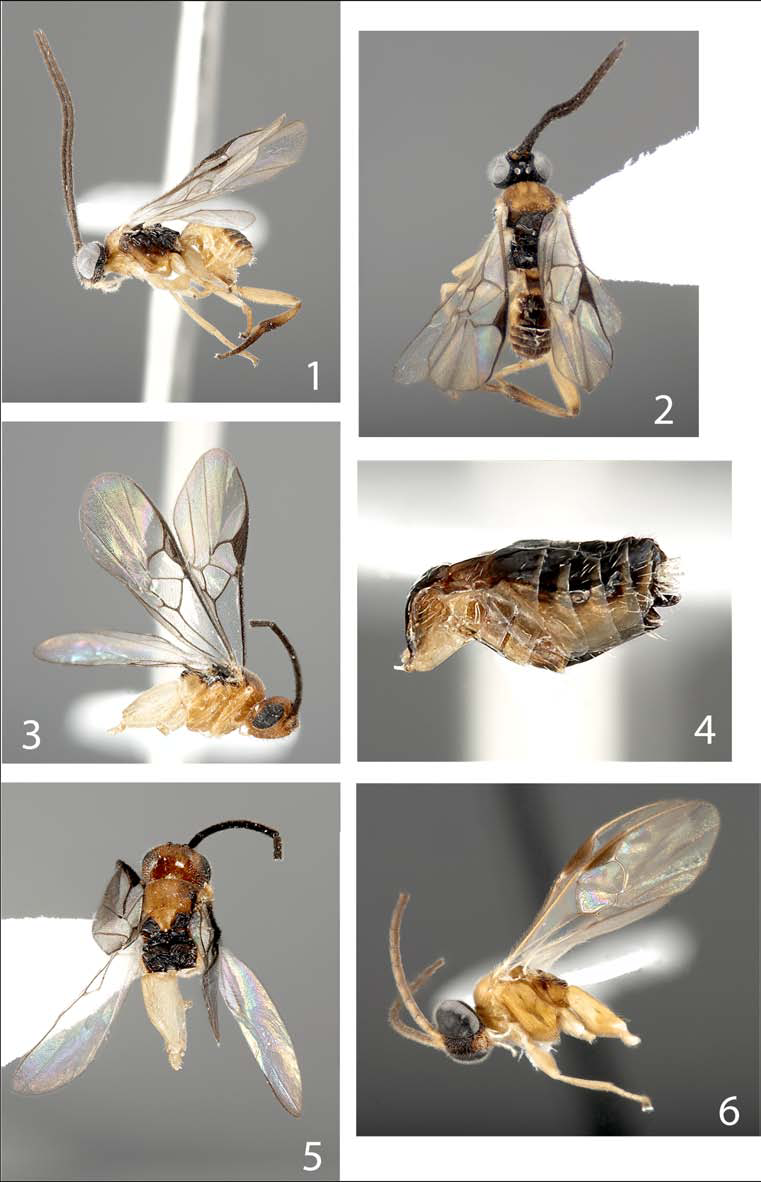
*Distatrix.* 1, *Distatrix loretta*, n. sp., lateral view of female. 2, dorsal view of female. 3, *Distatrix xanadon*., n. sp.. 3, lateral view of head, mesosoma and hind coxae. 4, lateral view of metasoma. 5, dorsal view of head, mesosoma and hind coxae. 6, *Distatrix vigilis,* n. sp., lateral view of head, mesosoma and hind coxae.

***Mesosoma*:** (Figures 1–2) pronotum and medial mesonotal lobe honey-orange, all ventral regions from the scutoscutellar scrobe to propodeum black. Pronotum and propleuron honey-orange except black dorsal and anteriad mesopleuron, lateral metanotum and metapleuron. Pronotum lacking dorsal groove. Mesoscutum evenly and shallowly punctate, surface otherwise polished; width at tegulae a little less than width of head. Scutoscutellar scrobe composed of eight confluent crenulations, very weakly arched medially. Scutellar disc subtriangular, wider anteriorly and tapering posteriorly; shallowly punctate to polished. Metanotum weakly retracted from scutellum; sublateral setiferous lobes not developed. Propodeum mostly polished to weakly punctate. Anterior submedial margins slightly convex. Posterior margin with minute carinae radiating from nucha. Propodeal spiracles large and pronounced.

***Legs*:** pale honey-orange except telotarsus of fore- and middle legs, hind legs pale orange except dark brown tarsus and apex of tibia. Hind coxae long, extending beyond forth metasomal tergite in dorsal view.

***Wings*:** (Figure 36) hyaline, proximal venation of forewing even brownish, anterior margin of stigma more darkly pigmented. Tegula honey-orange. 2r of forewing weakly arched, equivalent in length to ICU, meeting 2+3RS at acute angle marked by a knob. R1 is 1.3x as long as stigma. Stigma 2x as long as broad. RS+M faded and partly transparent, roughly equal in length to RS. Length of m-cu transparent, 2Cua faded, 2Cub transparent and very light brown. Hind wing with vannal lobe flattened to slightly concave subapically, with reduced fringe over flattened portion.

**Figures 7–13.**
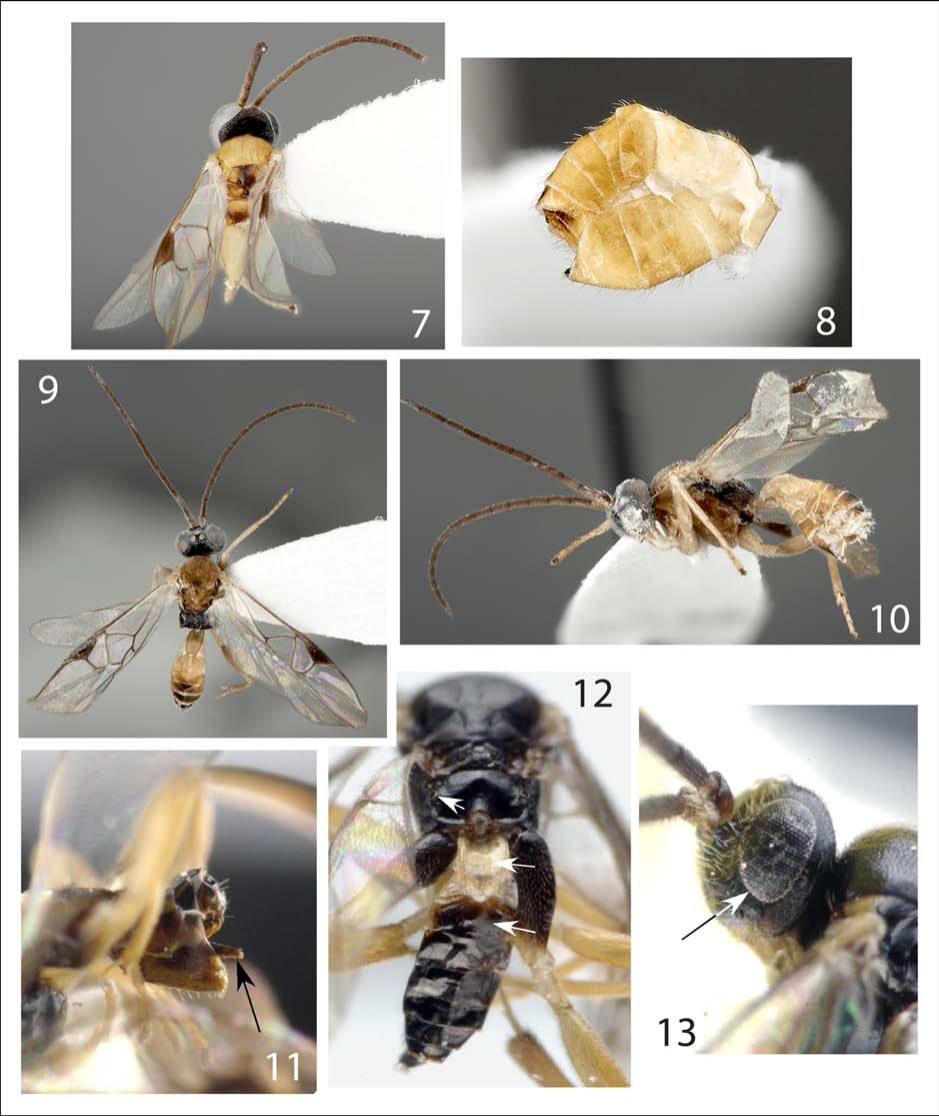
*Distatrix.* 7, *D*. *vigilis* dorsal view of head, mesosoma and hind coxae. 8, lateral view of metasoma. 9–10: *Distatrix pitillaensis,* n. sp.. 9, dorsal view. 10, lateral view. 11, *Distatrix antirrheae,* n. sp.. 11, lateral view of metasoma. 12, dorsal view of posterior mesosoma and metasoma. 13, lateral view of head.

***Metasoma*:** dorsal side dark brown except anterior and very posterior margin of tergite I. Lateral metasoma honey-orange, slightly darkening posteriorly. Ventral region and apex of hypopygium slightly darkened, ovipositor sheaths black with honey-orange apex. Tergite 1 very weakly sculptured, 2x as long as broad, barrel-shaped to very slightly narrowing posteriorly and forming concave margin with tergite II; broad excavation medially over anterior half, moderately arched in profile. Tergite II lightly sculptured, subtriangular, 2x broader posteriorly than long, posterior margin slightly concave; surface medially raised, defined by divergent groves more so anteriorly than posteriorly. Tergite III 1.4x as long as TII, medial region very slightly raised anteriorly. Remaining posterior terga of similar, unsculptured, overlapping form. Hypopygium short, evenly sclerotized, truncate at tip. Ovipositor sheath barely exerted from hypopygium, very few minute hairs at apex.

**Figures 14–19.**
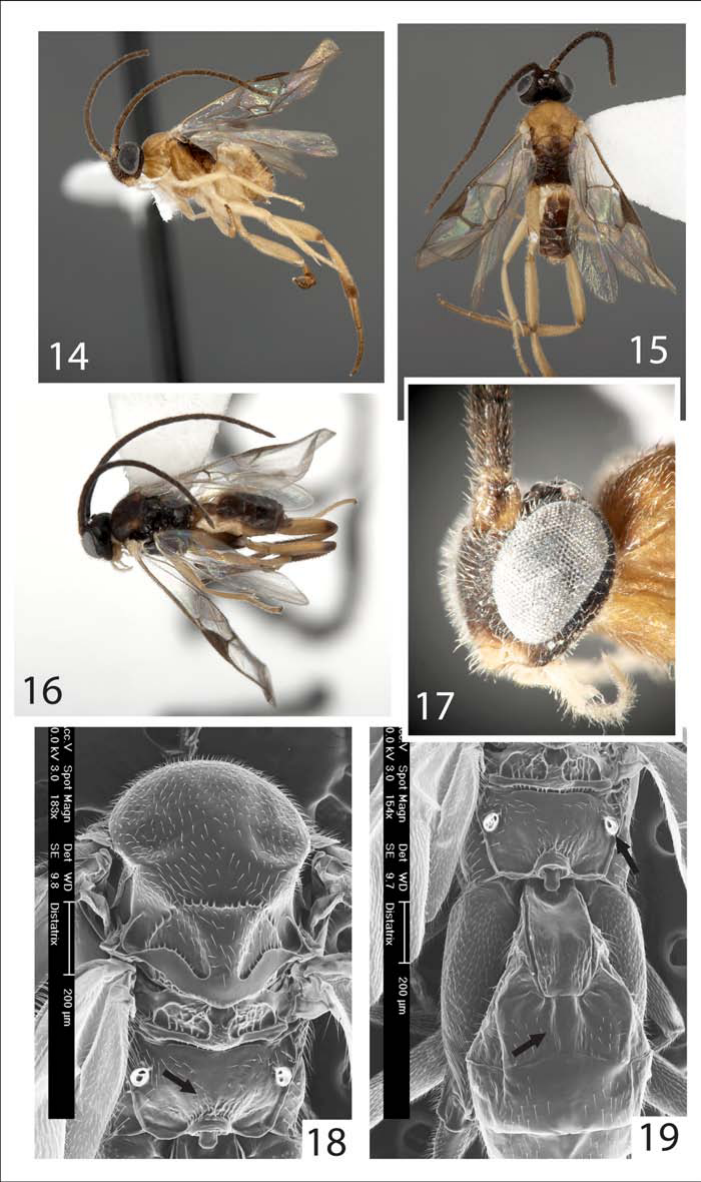
*Distatrix pandora,* n. sp.. 14, Lateral view of female. 15, Dorsal view female. 16, Lateral view male. 17, Lateral view of female head. 18, Dorsal view of mesosoma. 19, Dorsal view of propodeum and anterior three terga of metasoma.

**Figures 20–27.**
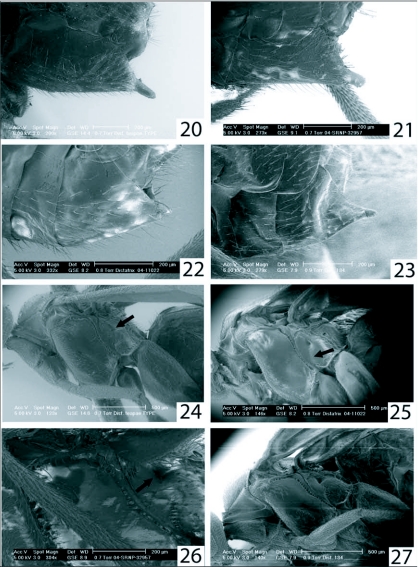
Hypopygia and lateral mesonota. 20, *Distatrix teapae* TYPE. 21, *Distatrix vigilis.* 22, *Distatrix pitillaensis.* 23, *Distatrix pandora.* 24, *Distatrix teapae* TYPE. 25, *Distatrix pitillaensis.* 26, *Distatrix vigilis.* 27, *Distatrix Pandora.*

***Males*:** unknown.

***Variation*:** undetermined.

**Figures 28–33.**
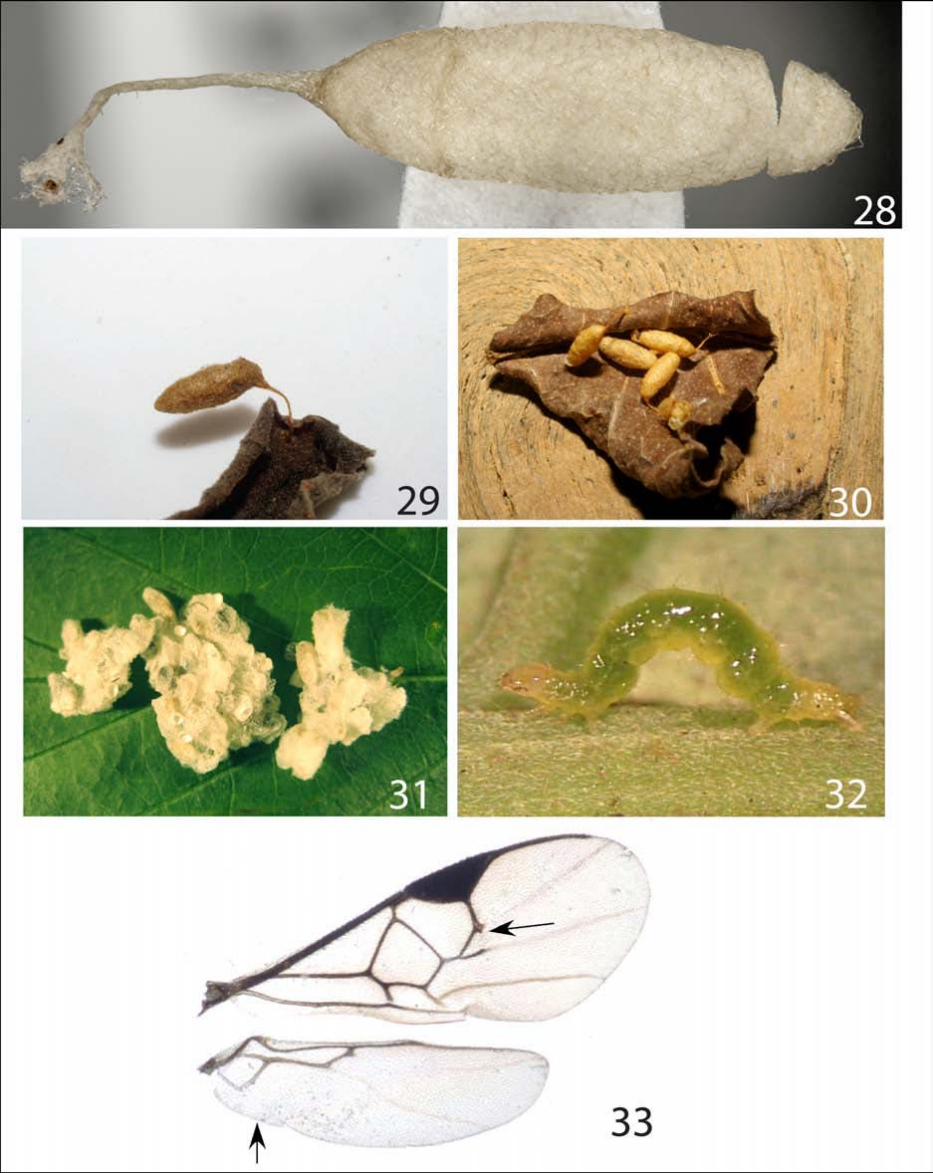
Cocoons (Image courtesy of DHJ). 28, *Distatrix pandora,* n. sp.. 29, *Distatrix pitillaensis,* n. sp.. 30, *Distatrix loretta,* n. sp.. 31, *Distatrix antirrheae,* n. sp.. 32, *Eois* *nympha.* (www.caterpillars.org). 33, *D*. *antirrheae,* wings.

***Cocoons*:** (Figure 30) gregarious, light brown in color, tough. Surface coarsely woven and attached to an asymmetrical silken stalk at one end to host plant substrate, stalk roughly 0.4x as long as cocoon. Wasp exited opposite end from stalk, leaving a partially detached lid.

### Material examined

Holotype female: Costa Rica: Guanacaste Province, Area de Conservatión Guanacaste, Sector Pitilla, Pasmompa, 440 meters. Latitude 11.01926, Longitude: -85.40997. 03 August 2005, Manuel Rios. Caterpillar Voucher: 05-SRNP-33043. Wasp Voucher: DHJPAR0004315. Deposited in U. S. National Museum, Washington, DC.

**Figures 34–36.**
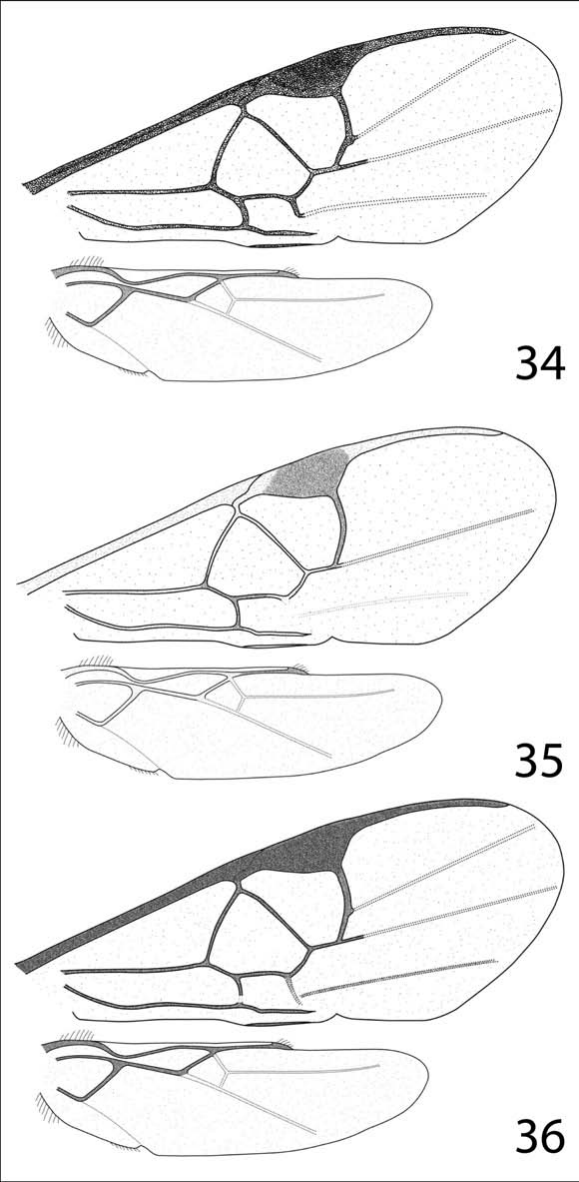
Wings. 34, *Distatrix pitillaensis* and *D*. *pandora.* 35, *Distatrix vigilis.* 36, *Distatrix loretta* and *D*. *xanadon.*

### Distribution

Known only from the type locality.

### Hosts

Five wasp larvae emerged from the single undetermined Geometridae caterpillar 98-SRNP-7305 found as a penultimate instar feeding on *Croton schiedeanus* Schlecht (Euphorbiaceae). The caterpillar was described as “green with two white lines down the back, with dots on them”. Cocoons formed on 18 August 2005, the wasps eclosed on 22 August 2005. Two *Distatrix* escaped during specimen transfer and two died and rotted after eclosion (Janzen & Hallwachs 2005).

### Etymology

The senior author chose the name *loretta* to honor his mother Loretta Grinter.

### Comments

This species is similar to *Distatrix pandora* in morphology and resembles *Distatrix xanadon* and *D. antirrheae* in having a higher proportion of black coloration. It has the same enlarged eyes as displayed by *D. belliger* (Wilkinson) and *D. solanae* Whitfield (females only at least in *D. loretta* and *D. solanae*). With *D. teapae* Nixon and at least eight other *Distatrix* species, *D. maia* Nixon, *D. formosus* (Wesmael) and *D. solanae* Whitfield, and all herein described new species (but not *D. belliger*), it shares a modified distal front tarsomere which is excavated apically on ventral side and bears a strongly curved, modified, spine.

The raised medial area of metasomal tergum II is defined by divergent groves that fade posteriorly. This, and a lighter and reduced yellow coloration, will differentiate this species from *pandora* or *teapae.* As with most other Neotropical species of this genus, the eyes of at least the females are enlarged, possibly suggesting a nocturnal or crepuscular host searching period; moderate xanthic coloration also suggests the latter. This species also shares the trait of gregariousness (having more than one parasitoid per host caterpillar) with *D. antirrheae* and these Old World species: *D. cerales* Nixon, *D. geometrivorus* (de Saeger), *D. anthedon* Nixon, *D. gratiosus* (Wilkinson) and *D. pallidocinctus* (Gahan); this is the only known species of gregarious *Distatrix* to spin pedunculate cocoons (other gregarious species spin loose cocoon masses or the cocoons stand up among host hairs) (Mason 1965).

### *Distatrix xanadon* Grinter, New Species (Figures 3–5, 36)

Holotype female. Body length 3.09mm; forewing length 3.27mm.

***Head*:** dark orange except for light yellow palpi, labrum and mandibles; interior margins of scape black, margins around ocelli darkened, black flagellomeres. Frons broad, barrel-shaped, slightly diverging both dorsally and ventrally due to shape of eyes, only slightly taller than broad, forming almost a square. Eyes of normal microgastrine size. Antennae slightly longer than forewing length, all but distal flagellomere with two ranks of placodes. Lateral ocellus separated from eye-margin by a distance equal to 1.5x its longer diameter.

***Mesosoma*:** (Figures 3, 5). honey-orange except dark black dorsal propodeum, metanotum, triangular polished areas, scutellar sulcus and mesonotal lobes adjacent to tegulae. Mesoscutum evenly and sparsely punctate, maximum width just less than width of head. Scutoscutellar scrobe comprised of an indeterminate number of confluent crenulations, weakly arched medially. Scutellar disc triangular, slightly longer than anteriorly broad, very shallowly punctate to polished. Metanotum evenly retracted from scutellum; sublateral setiferous lobes not developed, anterior margin of metanotum polished with few carinae. Propodeum weakly and evenly punctate, anteriorly convex, posterior steeply concave; short superimposed ridges posteriorly radiating from nucha.

***Legs*:** pale honey-yellow, except distal tarsomeres. Hind coxae long, extending beyond posterior margin of tergite III in dorsal view. Remaining legs missing from specimen, which is in poor condition.

***Wings*:** (Figure 36) hyaline, proximal venation of forewing even brown-black, distal venation darker. Setae become denser distally. Tegulae honey-orange. 2r of forewing arched, meeting 2 + 3RS at reduced knob. 2CUa very faded brown, curving apically away from union of 1m-cu & 1CU. 2Cub faded brown and very straight. Hind wing with vannal lobe reduced, flattened subapically and nearly denuded of setae, SC+R narrowing greatly after departing from apical edge of wing.

***Metasoma*:** (Figure 4) black dorsally except for honey-orange tergite II, laterally honey-orange except for black hypopygium, ovipositor sheath and dorsal margins. Tergite I very weakly sculptured and appearing polished, slightly widening ventrally then narrowing again slightly before meeting tergite II with broad excavation over anterior half, arched in profile; posterior end with a shallow and broad groove that does not extend anteriorly into excavation. Tergite II subtriangular, weakly sculptured as in tergite I; 2.0x broader posteriorly than maximum length. Medial surface raised slightly, more so anteriorly than posteriorly. Tergite III very broad, 1.2x as broad as tergite II. Remaining posterior terga of normal, unsculptured form. Hypopygium short, evenly sclerotized, truncate at tip. Cercus with a pronounced and dense cluster of setae. Ovipositor sheaths short and taper to blunt tip, weakly decurved, very few moderately long setae over exposed length.

***Males*:** unknown.

***Variation*:** undetermined.

***Cocoons*:** unknown.

### Material Examined

Holotype female: Costa Rica: Guanacaste Province, Area de Conservatión Guanacaste, Sector Cacao, Sendero Derrumbe. 1220 meters. Latitude: 10.92918 Longitude: -85.46426. 29 May 2007. H. Ramirez. Caterpillar Voucher: 06-SRNP-35250. Wasp Voucher: DHJPAR002071. Deposited in U. S. National Museum, Washington, D.C.

### Distribution

Known only from the type locality.

### Hosts

Reared from a single undetermined geometrid feeding on *Zanthoxylum melanostictum* Schltdl. & Cham. (Rutaceae). Caterpillar found as a second instar larva with “dark coffee color and coffee color head” (http://janzen.sas.upenn.edu). Cocoons formed on 11 June 2006 and eclosion on 25 June 2007.

### Etymology

The name *xanadon* is derived from Orson Welles's Xanadu; due to the unusual construction of the cocoon and high elevation from which it is known.

### Comments

This species of *Distatrix* is substantially different from other known Neotropical species and has normal microgastrine eye size in common with *D. antirrheae* and all eleven species of Old World described *Distatrix.* This species otherwise appears similar in morphology to *D. loretta* as described above. The overall black coloration, extremely arched profiles of propodeum and tergite I and larger setae along the ovipositor sheaths make this *Distatrix* unmistakable in comparison with other known Neotropical species. The normal microgastrine eye size suggests a closer relationship with Old World species of *Distatrix* or quite simply a diurnal host searching period. The superficial resemblance to *D. antirrheae* may also serve to predict the cocoon type (loosely woven, unstalked) for *D. xanadon.*

### *Distatrix vigllis* Grinter, New Species (Figures 6–8, 21, 25, 35)

Holotype female. Body length 2.91mm; forewing length 3.22mm.

***Head*:** deep brown-black except for honey-orange mandibles, clypeus, scape and pedicel. Palpi pale yellow to whitish. Flagellomeres honey-brown and darkening apically, all but distal flagellomeres with two ranks of placodes; antennae slightly longer than forewing length. Frons barrel-shaped due to size of eyes, dorsal crease notched apically. Eyes extremely large, forming most of visible portions of head in lateral view. Lateral ocellus separated from eye margin by extremely slim strip, nearly touching.

***Mesosoma*:** (Figures 6, 7, 25) mesoscutum honey-orange, scutellar disc medially dark brown, coloration continues dorsally onto medial metanotum. Lateral mesosoma honey-orange except around metapleural pit. Smooth groove leading up to lateral metapleural pit (Figure 25). Propodeum precisely honey-orange anteriorly and honey-brown posteriorly. Pronotum deeply grooved. Mesoscutum shallowly and evenly punctate, widest horizontal width just less than that of head. Scutoscutellar scrobe comprised of roughly ten confluent crenulations forming a lateral line across mesonotum. Scutellar disc subtriangular, narrowing posteriorly, very shallowly punctate. Metanotum weakly and evenly retracted from scutellum, sublateral setiferous lobes not developed. Metapleural pits weakly covered with crenulations. Propodeum very weakly punctate to smooth, evenly convex medially with short superimposed ridges radiating weakly from nucha, propodeal spiracles of normal size.

***Legs*:** pale honey-orange except very distal tarsomeres. Apices of femora and tibiae honey-orange. Hind coxae long, extending beyond posterior margin of tergite III. Remaining tibiae lost.

***Wings*:** (Figure 35) hyaline, proximal venation of forewing even light brown, medial regions of stigma more darkly pigmented, distal portion of C+SC+R and majority of R1, M + CU and 1-1A nearly white. Tegula honey-orange. Stigma 2x as long as broad. 2r of forewing arched, knob at 2 + 3RS junction absent, 3RSb absent. RS+M faded and partly white. 2Cua absent, m-cu meeting 1CUb at smooth angle, 2Cub transparent and very light brown. Hind wing with vannal lobe flattened to slightly concave subapically, with reduced fringe over flattened portion.

***Metasoma*:** (Figures 8, 21) tergite I–IV light honey-orange, posterior terga darkening, ovipositor nearly black with translucent apex. Lateral side of metasoma honey-orange. Tergite I very weakly sculptured, weakly punctate to smooth, 2x as long as broad at widest point, narrowing posteriorly and forming rounded end, broad steep excavation over anterior half, weakly arched in profile. Tergite II also weakly sculptured, weakly and evenly punctate, subrectangular with surface raised medially forming a “pinched” look that narrows anteriorly to meet with tergite I. Raised central area forming subtriangular medial region. Tergite III and posterior terga of normal, unsculptured, overlapping form. Hypopygium short, decurved, and tapering towards blunt tip, ovipositor sheaths with very few minute setae (Figure 21).

***Males*:** unknown.

***Variation*:** undetermined.

***Cocoons*:** unknown.

### Material examined

Holotype female: Costa Rica: Guanacaste Province. Area de Conservación Guanacaste, Sector Santa Rosa, Cerco Piedra. 285 meters. Latitude: 10.83085, Longitude: -85.61778. 13 June 2004. Freddy Quesada. Caterpillar Voucher: 04-SRNP-11022. Wasp Voucher: DHJPAR0012024. Deposited in U. S. National Museum, Washington, D.C.

### Distribution

Known only from the type locality.

### Hosts

Reared from a single Noctuidae, *Isogona* sp. (Noctuidae; Catocalinae), found feeding on *Celtis trinervia* Lam. (Celtidaceae) as a pen-pen-penultimate instar. Cocoons formed on 20 June 2004 and eclosion on 23 June 2004.

### Etymology

This species is named for its unusually large eye size.

### Comments

This species closely resembles *Distatrix teapae* Nixon, sharing with it and *D. belliger* (Wilkinson), *D. loretta, xanadon, pitillaensis, pandora,* the enlarged eyes (females only at least in *D. loretta, D. pitillaensis, D. pandora* and *D. solanae*). With *D. teapae* and at least all other described species herein and *D. maia* (Nixon) and *D. formosus* (Wesmael) (but not *D. belliger*), it shares a modified distal front tarsomere, which is excavated apically on the ventral surface and bears a strongly curved, modified, spine.

The new species differs from *D. teapae* Nixon, *D. pitillaensis* and *D. pandora* most notably in wing venation, coloration, propodeal morphology and placement of antennae on the head. In the new species the wing venation is reduced in both structure and coloration with the absence of 2Cua and 3RSb and nearly white regions of M+CU, 1-1A, C+SC+R and R1 (Figure 35). The antennae are inserted on the head markedly lower than any examined species so far. There is also a distinct absence of crenulations on the metapleuron and mesopleuron, the grove leading up to the lateral metapleural pit is smooth, the sternaulus is absent and propodeal spiracles are small to normal in size (Figure 25). In *D. teapae* and *D. pitillaensis* and *D. pandora* the lateral metapleural groove is pronounced, the sternaulus is present and spiracles are large and pronounced. The new species is also highly xanthic in coloration, suggesting a nocturnal host searching period, similar to that of *D. teapae* and other Neotropical *Distatrix.*

### *Distatrix pitillaensis* Grinter, New Species (Figures 9–10, 29, 22, 26, 34)

Holotype female. — Body length 3.45mm; forewing length 3.74mm.

***Head*:** black except honey-orange mandibles, clypeus and scapes. White-yellow palpomeres, labrum, palpi and dark orange to brown flagellomeres that darken dorsally along length. Frons medially taller than broad due to size of eyes, barrel shaped to broadening dorsally, evenly punctate. Eyes extremely large and forming most of visual portion of head from lateral view. Ocellus forming equilateral triangle, distance to eye-margin equal to less than one half of the longest length of lateral ocelli. Antennae slightly longer than forewing length, tapering apically, all but distal flagellomeres with two ranks of placodes.

***Mesosoma*:** (Figures 9, 10, 22, 26) honey-orange except posterior metanotum excluding post scutellar depression, dorsal region of mesopleuron and lateral metanotum. Propodeum black. Mesoscutum evenly, sparsely and shallowly punctate; tapering medially into a shallow excavation before reaching the scutoscutellar scrobe which is comprised of an indeterminate number of confluent crenulations. Scutellar disc strongly arched medially, very weakly punctate to polished, subtriangular, anterior width just less than equal to vertical length. Metanotum evenly retracted from scutellum; sublateral setiferous lobes not developed. Post scutellar depression pronounced, posterior carina forming a broad “U” shape. Propodeum weakly punctate to smooth, evenly convex medially, with short superimposed ridges weakly radiating posteriorly from nucha. Propodeal spiracles large and pronounced.

***Legs*:** honey-orange except tarsal claws. Hind coxae short, 0.5x as long as hind femur, barely extended half way along length of tergite 1. Hind tibia and tarsi defined by rows of short orange spines, subequal in length with normal setae but thicker. Inner apical spurs of hind tibia 2.0x as long as outer.

***Wings*:** (Figure 34) hyaline, proximal venation of forewing even brownish, anterior margin of stigma more darkly pigmented. Tegula honey-orange. 2r of forewing weakly arched, meeting junction of 2 + 3RS at distinct but not acute angle marked by knob. R1 is 1.3x as long as stigma. Stigma 2x as long as broad. RS+M faded and partly transparent. 2Cua faded, 2Cub transparent and very light brown. Hind wing with vannal lobe flattened to slightly concave subapically, with reduced fringe over flattened portion. *Metasoma.* (Figure 22) — Light honey-orange except darkened dorsal regions from terga IV to basal tergites. Hypopygium and ovipositor sheaths light brown to light yellow apically. Terga I very weakly sculptured, 2.2x as long as broad at broadest point, subparallel-sided to narrowing posteriorly then forming a square angle with posterior margin, with broad excavation medially over anterior half; moderately arched in profile. Terga II moderately sculptured, subtriangular, 2.0x broader than posteriorly than maximum length, with medial concave posterior margin; surface raised medially to form a parallel sided convex region running length of terga. Terga III and posterior terga of normal, unsculptured, overlapping form. Hypopygium of medium length and truncate at tip. Ovipositor sheaths short, barely exerted from hypopygium and evenly tapering towards blunt tip, armed with minute setae along tip (Figure 22).

***Males*:** body length: 2.83mm, forewing length: 3.05mm. Head blackish, anterior mesoscutum dark orange, propodeum black. Eyes of normal microgastrine proportions, with genae and postgenae clearly visible in lateral view. Anterior half of metasoma light yellow darkening black towards posterior. Hind coxae of normal microgastrine proportion, extending past second metasomal tergite.

***Variation*:** undetermined.

***Cocoons*:** (Figure 29) dark brownish tan with roughly woven surface and attached by an asymmetrical silken stalk to the tip of the host plant leaf.

### Material Examined

Holotype female: Costa Rica: Guanacaste Province. Area de Conservación Guanacaste, Sector Pitilla, Sendero Laguna, 680 meters. Latitude: 10.9888, Longitude: -85.42336. 22 May 2004, C. Moraga. Caterpillar Voucher: 04-SRNP-32957. Wasp Voucher: DHJPAR0012025. Deposited in U.S. National Museum, Washington, D.C. Paratypes: 1 male, COSTA RICA: Guanacaste Province. Area de Conservación Guanacaste, Sector Pailas, Estación Pailas, 785 meters. Latitude: 10.76969, Longitude: -85.34080. 23 July 1998, D.H. Janzen. Caterpillar Voucher: 98-SRNP-11498. Wasp Voucher: DHJPAR0005175. Deposited in U.S. National Museum, Washington, D.C.

### Distribution

This species is known from two locations in Guanacaste province of northwestern Costa Rica ranging from 680–780 meters. Both inhabit upper elevation moist forest and should be sought at similar elevations in Central America where the host caterpillar is known.

### Hosts

Female reared from a single *Cimicodes albicosta* (Geometridae: Ennominae), found feeding on *Siparuna thecophora* Poepp. & Endl (Monimiaceae) on 22 May 2004, cocoon formed on 28 May 2004 and eclosed on 6 June 2004. Male reared from a single indeterminate *Geometridae* “same as 98-SRNP-11500” feeding as pen-penultimate instar on *Croton morifolius* (Euphorbiaceae) on 23 July 1998.

### Etymology

The name of this species is derived from the type locality in Costa Rica.

### Comments

This species most closely resembles *Distatrix teapae* in coloration, sharing with it and *D. belliger* (Wilkinson), *D. loretta, xanadon, vigilis* and *pandora* the enlarged eyes (females only). With *D. teapae* and at least the other species described herein, and excluding *D. belliger,* it shares a modified distal front tarsomere, which is excavated apically on the ventral side and bears a strongly curved, modified, spine.

This new species differs from *D. teapae* most strikingly in the hind coxae of the female, showing a vastly reduced length that barely extends beyond tergite I (as in the corresponding male and all discussed *Distatrix* and *D. teapae* the hind coxae is large, extending beyond the third tergite). *D. pitillaensis* is also darker in coloration with a more dark honey-orange mesosoma and metasoma with dark brown margins.

### *Distatrix pandora* Grinter, New Species (Figures 14–19, 23, 27, 28, 32, 34)

Holotype female. — Body length 2.62mm; forewing length 2.90mm.

***Head*:** (Figure 17) black except white-orange palpi, yellow-orange scapes, pedicels, labrum and clypeus; mandibles white-yellow and darkening to orange apex; face medially honey-orange; flagellomeres dark brown. Frons subrectangular and nearly as tall as broad, evenly and weakly punctate, anterior tentorial pits pronounced and rimmed with black. Eyes extremely large, forming most of visible portions of head in lateral view. Antennae slightly longer than forewing length and tapering gradually; all but distal flagellomere with 2 ranks of placodes. Lateral ocellus separated from eye-margin by a distance equal to ¾ its longer diameter. Flagellomeres separated from a distance equal to one flagellomere width.

***Mesosoma*:** (Figures 14–19, 27) honey-orange except light brown medial section of scutellar disc, triangular polished areas; dorsal metanotum and propodeum brown; extreme ventral and dorsal portions of mesopleuron and majority of metapleuron and lateral metanotum light brown. Mesoscutum evenly and weakly punctate to slightly polished, broadest width across mesoscutum barely less than that of head. Scutoscutellar scrobe comprised of indeterminate number of confluent crenulations, evenly horizontal across mesoscutum. Scutellar disc slightly longer than anteriorly broad, distinctly and shallowly punctate. Metanotum weakly and evenly retracted from scutellum; sublateral setiferous lobes not developed; axillary troughs posteriorly marked with minute intruding triangular notch. Mesopleural sternaulus comprised of seven confluent crenulations. Metapleuron posterodorsally bordered with 3–4 confluent crenulations. Propodeum moderately punctate to smooth, evenly convex medially, with very short superimposed ridges radiating medially from nucha (Figure 18).

***Legs*:** pale honey-orange except very distal tarsomeres of pro-and mesothoracic legs; hind leg darkens at apical tip of femur and tibia, remaining tarsi brown, telotarsi on all legs nearly black. Foreleg with modified spur medioventrally on distal tarsomere. Hind coxae long, extending to just beyond posterior margin of third metasomal tergum. 25–30 spines on outer faces of hind tibiae, subequal in length with normal setae but thicker and pale golden. Inner apical spurs of hind tibiae 1.3x as long as outer, about 0.8x length of hind basitarsus.

***Wings*:** (Figure 34) hyaline, proximal venation of forewing even brownish, anterior margin of stigma more darkly pigmented. Tegula honey-orange. 2r of forewing weakly arched, meeting junction of 2 + 3RS at distinct but not acute angle marked by knob. R1 is 1.3x as long as stigma. Stigma 2x as long as broad. RS+M faded and partly transparent. (RS + M)a very thin, junction with (RS + M)b faded and nearly transparent. 2Cua faded, 2 Cub transparent and very light brown. Hind wing with vannal lobe flattened to slightly concave subapically, with reduced fringe over flattened portion.

***Metasoma*:** (Figures 19, 23) honey-orange except brown dorsal surfaces of tergite II and remaining posterior terga; tergite II medially lighter along convex ridge. Tergite I light honey-yellow. Hypopygium evenly honey-orange, ovipositor sheaths darker orange. Tergite I weakly sculptured with faint medial grove running length, 2.0x as long as broad at broadest point, subparallel sided and narrowing posteriorly then rounding at posterior end, with broad excavation medially over anterior half; moderately arched in profile. Tergite II very weakly sculptured to polished, sub triangular, 2.0x broader posteriorly than maximum length, shortest medially with abruptly raised center; raised medial area defined by anterior grooves diverging at an angle close to 90°, which then converge again posteriorly to form a weak diamond shape (Figure 19). Tergite III and posterior terga of normal, unsculptured, overlapping form. Hypopygium relatively short, evenly sclerotized, truncate at trip; lateral edge evenly and sparsely covered with setae. Ovipositor sheaths short, truncate at tip and weakly decurved, armed with very few minute hairs along apex (Figure 23).

***Males*:** (Figure 16) body length: 2.72mm, forewing length: 2.78mm. Mesosoma and metasoma mostly blackish. “Shoulders” of lateral mesonotal lobes tipped in orange. Eyes of normal microgastrine proportion. Flagellomeres conspicuously longer than forewing length. Genae and postgenae visible in lateral view.

***Cocoons*.** (Figure 28). Light tan, roughly woven, tough. Attached to host substrate by asymmetrical silken stalk at opposite end from parasitoid eclosion. In a few instances the wasp emerged from the same side as the silken stalk.

### Variation

There is some degree of variation in deepness of orange coloration throughout the specimen, perhaps varying geographically.

### Material Examined

Holotype female: PANAMA: Barro Colorado Island. 9°09′N, 90°51-W, artificial island made up of 15km2 of lowland moist forest located in the Panama Canal (Gatun Lake), 2003. Paratypes: 9 females, 10 males, similar data except emergence and pupation dates. 9 females, 10 males, similar data except 2004. 1 female, similar data except 11 June 2001. 1 male, 1 female, similar data except 22 July 2003. 3 females, 3 males, similar data except 23 July 2003. 1 female, similar data except 25 June 2005. (MIUP, CCG collection). 3 females, 7 males: COSTA RICA: Heredia Province. La Selva Biological Reserve, located at 100m on the Caribbean slope, 10°26′N 83°59′W (Hartshorn and Hammel 1994, http://www.ots.duke.edu/en/laselva/intro.shtml). 1 male, ECUADOR: Napo Province, Yanayacu Biological Station and Center for Creative Studies, 5 km W. Cosanga, 2100m, 0°42′01.33″S, 77°44′00.00″W, 16 March 2002. 1 male, similar data except 3 June 2001.

### Distribution

*D. pandora* is primarily known from lowland moist forest in Panama and Costa Rica, ranging from 50–100m. However, two specimens were reared from highland cloud forest (80% primary forest) in Ecuador at 2100m, indicating a surprisingly diverse and large range extension.

### Hosts (Figure 32)

Single holotype female were reared from *Eois nympha* (Geometridae) feeding on *Piper cenocladum* C. DC. (Piperaceae). Hosts reared from Panama are from an undetermined species of *Eois* feeding on *Piper aequale* Vahl and *Piper scheideanum* Steud.. Two male specimens from Ecuador reared from an undetermined Geometridae.

### Etymology

This species is named *pandora* because of the confusion between this and a similar species of *Diolcogaster* for which it may be mistaken.

### Comments

This species is almost identical to *Distatrix teapae* (Nixon), both in morphology and coloration, diverging only with the size of the lateral metapleural pit and angle of the lines on metasomal tergum II; sharing with it and *D. solanae* Whitfield, *D. xanadon, pitillaensis* and *D. belliger* (Wilkinson) the enlarged eyes (females only at least in *D. pandora*). With *D. teapae* and at least *D. maia* (Nixon), *D. formosus* (Wesmael), *D. loretta, xanadon, vigilis, pitillaensis, antirrheae* (but not *D. belliger*), it shares a modified distal front tarsomere, which is excavated apically on the ventral side and bears a strongly curved modified spine.

This new species differs from *D. teapae* in having an overall smaller body size, lighter orange coloration, and these slight morphological differences: Metasomal tergum II with anterior medial area defined by grooves diverging at an angle roughly 90°, posteriorly converging and forming a weak diamond shape, whereas in *D. teapae* the angle is greater than 120° (Figure 19). The hypopygium of the new species appears to be wider medially, more so than immediately anteriad sternum (in *D. teapae* the hypopygium gradually tapers towards anterior apex (Figure 20). The new species also is conspicuously setiferous along the entire width of ventral sclera, whereas in *D. teapae* the setae are constricted to the ventral third of the specimen. The distances between the ocellus and eye margins, as well as the flagellomere distances, are slightly larger by about half than that of *D. teapae. D. pandora* also shares with at least herein described species, a very large lateral metapleural pit, which appears to be reduced in *D. teapae* (Figure 24). Mesopleural sternaulus is also nearly absent in *D. teapae.*

This species also closely resembles another undescribed species of microgastrine wasp in the genus *Diolcogaster* that shares similar coloration and a shortened ovipositor. Separating the species is easily done if the cocoons are associated with the specimen. The similar *Diolcogaster* spins an unstalked cocoon with petal like layers that curl outward, whereas the Distatrix will always have a stalked, tightly woven, cocoon. When no cocoon is present a close examination of the eye size, propodeal sculpturing, propodeal spiracles or hind coxae will quickly delimit the species.

*D. pandora* appears to be a specialist on *Eois* caterpillars, however specific level interactions are undetermined.

### *Distatrix antirrheae* Whitfield & Grinter, New Species (Figures 11–13, 31, 33)

Holotype female. Body length 2.5mm; forewing length 2.9mm.

***Head*:** black except yellow-orange palpi, and honey-orange labrum. Scape medium brown with darkened edges, flagellomeres dark brown. Frons broader that medially tall, weakly punctate. Compound eyes of normal microgastrine size (Figure 32). Antennae slightly longer than forewing length and tapering gradually towards apex; all but distal flagellomeres with two ranks of placodes. Lateral ocelli slightly farther apart from each other than width of median ocellus.

***Mesosoma*:** (Figures 12, 13) mesosomal color entirely black except tegulae and most of legs. Pronotum mostly polished laterally and lacking dorsal groove. Mesoscutum evenly and shallowly punctate (especially posteriorly), surface otherwise polished; width at tegulae more or less equal with width of head. Scutoscutellar scrobe composed of six confluent crenulations, very weakly arched medially. Scutellar disc subtriangular, longer than anteriorly broad; indistinctly punctate to polished. Metanotum weakly retracted from scutellum; sublateral setiferous lobes not developed. Propodeum mostly polished throughout, with anterior submedial margins slightly convex, and posterior margin with minute carinae radiating from nucha. Propodeal spiracles slightly enlarged (Figure 12).

***Legs*:** pale honey-orange throughout except mostly blackish hind coxae and apex of hind tibiae. Hind coxae long, extending to end of third metasomal tergite in dorsal view.

***Wings*:** (Figure 33) hyaline with slight brownish tinge, proximal venation of forewing even brownish, anterior margin of stigma more darkly pigmented. Tegula translucent pale yellowish. 2r of forewing nearly straight, meeting 2RS at acute angle marked by a prominent squarish knob that suggests junction with spectral r-m vein (Figure 33). R1 1.3x as long as stigma, which is 2x as long as broad. RS+M less strongly pigmented, m-cu, 2Cua and 2Cub pigmented light brown. Hind wing with vannal lobe flattened subapically, with reduced fringe over flattened portion (Figure 33).

***Metasoma*:** dorsal side dark brown to black except whitish yellow tergite I and its laterotergites (Figure 12). Ventral region of metasoma dark brown to black except lighter anterior sternites. Tergite 1 very weakly sculptured, 2x as long as broad, evenly narrowing posteriorly to a round apex at junction with tergite II, broadly excavated medially over anterior half, moderately arched in profile. Tergite II lightly sculptured, with diverging anterior grooves and concave posterior margin suggesting chevron-shaped region that is raised medially. Tergite III longer than TII, especially medially. Remaining posterior terga of similar, unsculptured, overlapping form. Hypopygium short, evenly sclerotized, truncate at tip (Figure 11). Ovipositor sheath barely exerted from hypopygium, with very few minute hairs at apex, appearing hairless (Figure 11).

***Males*:** similar to females except with lateral margins of metasomal tergite I darkened.

***Cocoons*.** (Figure 31). Gregarious, whitish, loosely woolly and forming irregular piles.

### Variation

All known specimens were from the same host caterpillar and are highly similar.

### Material examined

Holotype female: Ecuador: Napo Province, Yanayacu Biological Station and Center for Creative Studies, 5 km W. Cosanga, September 2007 (adults eclosed 16 Nov. 2007), 00° 35.949S 77°53.403W, 2100m, rearing number 26548. Paratypes: 34 other females and 2 males, same data. Holotype and 20 paratypes deposited in U. S. National Museum, Washington, DC; other paratypes in Illinois Natural History Survey, Champaign, IL.

### Distribution

Known only from type locality. Parasitoid may be present in populations of the nominate subspecies of *Antirrhea adoptiva* in Colombia; and quite possibly in other species throughout the genus *Antirrhea* for which the biology is poorly known (Greeney et al. this issue).

### Hosts

The gregarious brood of parasitoids emerged from a caterpillar of *Antirrhea adoptiva porphyrosticta* (Watkins), a rarely collected nymphalid feeding on *Chusquea scandens* Kunth (Poaceae). For additional details on this host and its biology, see Greeney et al. (this issue). In addition to the holotype and paratypes mentioned above, approximately 10 other adult wasps failed to eclose from the cocoon mass, so the brood size appears to have been around 45.

### Etymology

The species is named after the genus of its host caterpillar.

### Comments

This species can be easily distinguished from its other known congeners in the New World by its combination of a whitish and posteriorly evenly narrowing first metasomal tergite and otherwise quite dark body, along with the normal-sized eyes in both sexes and the suggestion of a forewing areolet. The gregarious habit, host family and unstalked, wooly white cocoons are also distinctive among New World species, but would not be out of place among some Old World species.

### Editor's note

Paper copies of this article will be deposited in the following libraries. Senckenberg Library, Frankfurt Germany; National Museum of Natural History, Paris, France; Field Museum of Natural History, Chicago, Illinois USA; the University of Wisconsin, Madison, USA; the University of Arizona, Tucson, Arizona USA; Smithsonian Institution Libraries, Washington D.C. USA; The Linnean Society, London, England.
